# A longitudinal study of loneliness in autism and other neurodevelopmental disabilities: Coping with loneliness from childhood through adulthood

**DOI:** 10.1177/13623613231217337

**Published:** 2023-12-28

**Authors:** Hillary Schiltz, Dena Gohari, Jamie Park, Catherine Lord

**Affiliations:** University of California, Los Angeles, USA

**Keywords:** autism, coping, lifespan, loneliness

## Abstract

Many autistic people and people with non-spectrum neurodevelopmental disabilities (e.g. intellectual disability) report feeling lonely, which can negatively impact their well-being. There is little longitudinal research, however, tracking changes in how autistic people experience, conceptualize, and cope with loneliness throughout their lives. A longitudinal sample of 114 people, which included autistic participants and participants with neurodevelopmental disabilities, characterized experiences of loneliness, perceptions of other people’s loneliness, and strategies used to cope with loneliness from childhood to adulthood. Level of loneliness and coping strategies were coded from Autism Diagnostic Observation Schedule Modules 3 and 4 protocol forms. Autism Diagnostic Observation Schedule Loneliness Ratings were correlated across time and increased from adolescence to young adulthood. The most common loneliness coping strategies were Behavioral Distraction (e.g. watching TV) and Instrumental Action (e.g. seeking social contact), which were both used by more people in adulthood than childhood. Those who used Behavioral Distraction and a greater number of coping strategies had higher Autism Diagnostic Observation Schedule Loneliness–Self Ratings (i.e. were lonelier) during adolescence and adulthood. Findings highlight adulthood as a particularly vulnerable time for loneliness and indicate a need for more support and social opportunities for autistic adults and adults with neurodevelopmental disabilities who wish to make more social connections.

**Lay abstract:**

We know that many autistic people feel lonely, but we don’t know whether their loneliness changes over time. Our research study followed autistic people and people with other non-spectrum neurodevelopmental disabilities from childhood through young adulthood and asked them about their loneliness. While many people told us they felt lonely or very lonely, a sizable group also told us that they do not feel lonely. We found that people who reported feeling lonely earlier in life were likely to also report feeling lonely later in life. Overall, autistic people and people with other neurodevelopmental disabilities in our study became lonelier from adolescence to adulthood. People described multiple ways they cope with feeling lonely, such as distracting themselves or reaching out to connect with another person. People who used distraction tended to be lonelier than those who did not. Our findings tell us that there is a need for greater support of social connections for many autistic people as they become adults.

Many autistic youth and adults and people with other neurodevelopmental disabilities (NDDs) report feeling lonely (Alexandra et al., 2018; Deckers et al., 2017; Ee et al., 2019). Loneliness—a negative emotional experience related to a perceived discrepancy between actual and desired social connections (Peplau, 1982)—can have negative effects on mental and physical well-being (Baczewski & Kasari, 2021; Hedley et al., 2018; Hymas et al., 2022; Park et al., 2020; Schiltz et al., 2021) and life satisfaction (Feldhaus et al., 2015). While there is growing research on rates and correlates of loneliness in autism (see Umagami et al., 2022) and other NDDs (Alexandra et al., 2018; Gilmore & Cuskelly, 2014), many questions remain. Specifically, considering the developmental nature of autism and other NDDs and the significant impact loneliness can have on adult outcomes (Alexandra et al., 2018; Umagami et al., 2022), research is still needed to better understand potential developmental changes in loneliness from childhood into adulthood. Therefore, this study aims to provide insights into the ways autistic people and those with related NDDs experience, conceptualize, and cope with loneliness over time.

A growing body of research has focused on cross-sectional levels and correlates of loneliness in autistic people (Hymas et al., 2022; Umagami et al., 2022) and people with other NDDs (Alexandra et al., 2018; Emerson et al., 2021; Pagan, 2020). For example, cross-sectional studies have identified associations between social communication differences and loneliness in autistic adults (Han et al., 2019; Schiltz et al., 2021). As such, it has been posited that social communication challenges may lead to unsuccessful social experiences in a non-autistic majority society, which yield feelings of disconnection and loneliness (Schiltz et al., 2021). Notably, social and societal context matter; social opportunities may be limited in this population, particularly in adulthood, due to factors such as unemployment or living arrangement (Emerson et al., 2021; Pagan, 2020), and social belonging for autistic people can be impeded by a lack of autism acceptance and understanding on behalf of others (Hwang et al., 2017). Consistent with these notions, meta-analytic evidence indicates higher levels of loneliness in autistic compared to non-autistic people (Hymas et al., 2022), which includes studies of youth (Bauminger et al., 2003; Bauminger & Kasari, 2000), adolescents (Lasgaard et al., 2010; Locke et al., 2010), and adults (Ee et al., 2019), although not all studies of children have found this group difference (Chamberlain et al., 2007). Because autism and other NDDs are, for the most part, lifelong conditions, longitudinal studies are needed to build on these cross-sectional age-related findings to determine whether loneliness is more likely to occur in certain developmental stages and whether those who are lonely in childhood are likely to be lonely as adults.

Childhood, adolescence, and adulthood each come with their own social restructuring and life events—changes that may impact loneliness (Buecker et al., 2021). For autistic people, challenges navigating developmental transitions may be compounded by difficulties inherent to autism (e.g. social communication differences; Anderson et al., 2018; Picci & Scherf, 2015). This may be especially true across the transition to adulthood, when social structures and resources that are integrated into school systems no longer provide support (Shattuck et al., 2012, 2020). In the general population, meta-analytic data have shown age-related changes in loneliness; average levels of loneliness have been found to decrease throughout childhood (6–12 years old) and remain relatively stable from teen to old age (through 80 years old) (Mund et al., 2020). In addition, the consistency in people’s reports of their level of loneliness (i.e. correlations across time) increases across childhood and adolescence (6–12 years), is relatively high in middle-adulthood (40.1–60 years), and becomes less stable into older age (60.1 years and older) (Mund et al., 2020). Research has also found that for a subset of the general population, loneliness remains chronically high or increases from childhood into adulthood (ages 7 through 20)—a pattern that is linked to poorer physical and psychological functioning (van Dulmen & Goossens, 2013).

In addition to differences in the level of loneliness among autistic people and people with NDDs, there may be distinct ways loneliness is experienced and conceptualized. Studies of youth (8–14 years old) have indicated that the difference between feeling lonely and being alone may be particularly striking among autistic people compared to non-autistic people (Bauminger & Kasari, 2000, 2002). That is, there is some evidence that being alone is less tightly linked with feeling lonely in this population (Bauminger & Kasari, 2000, 2002). Similarly, qualitative research has revealed that some autistic adults describe being on the “outside” of social experiences leading to feelings of boredom instead of loneliness and having a *greater need* for social isolation than non-autistic adults due to emotional and cognitive strain from everyday activities (Elmose, 2020). Because of these potential differences in how loneliness is experienced by autistic people, the perception of others’ loneliness may likewise differ, as well. Previous research indicates that in the general population, most people tend to think they are lonelier than other people (Cacioppo & Hawkley, 2005; Duck et al., 1994; Haslam, 2022; Spithoven et al., 2017; Vanhalst et al., 2013, 2015), yet it is unknown whether the same phenomenon occurs for autistic people.

Perhaps just as relevant as the experience of loneliness is how people cope with feelings of loneliness. Loneliness has been compared to a “thirst”—signaling the need to seek social contact, as a thirsty person might seek water (Cacioppo & Cacioppo, 2018). However, people who feel lonely often behave in ways that perpetuate rather than reduce their loneliness (e.g. withdrawing from others; J. T. Cacioppo & Cacioppo, 2018; Vanhalst et al., 2015). Empirical studies have identified many coping responses in non-autistic populations specific to loneliness that vary widely in terms of person of focus (self or other) (Vaarala et al., 2013), degree of helpfulness or adaptiveness (Rokach, 1990), intensity of activity (Schoenmakers et al., 2012), order of use (first or second, etc.) (Rokach, 1990), and mode of expression (thoughts or behavior) (Vaarala et al., 2013). In addition, some studies in the general population have classified coping responses to loneliness based on *Families of Coping* identified in the broader coping literature (e.g. Accommodation, Escape, Problem-Solving; Besevegis & Galanaki, 2010; Skinner et al., 2003; Vasileiou et al., 2019).

Certain Families of Coping are thought to be relatively more adaptive than others, namely with respect to three factors: (1) their long-term developmental consequences, (2) their subjective experience, and (3) their current qualities. For example, social withdrawal or isolation is regarded as maladaptive given the potential for exacerbating the risk factor (e.g. social withdrawal may perpetuate feelings of loneliness by further preventing the individual’s engagement with social opportunities). Furthermore, maladaptive coping strategies that contribute to harsh engagements with the self (e.g. self-blame and submission) can contribute to lower self-efficacy, confidence, and sense of perceived control (Skinner et al., 2003). Maladaptive coping strategies (e.g. disengagement, avoidance, distraction) have been linked with mental health difficulties, including higher levels of psychological distress, anxiety, and depression in both autistic (Khor et al., 2014; Muniandy et al., 2021, 2022) and non-autistic (Compas et al., 2017; Nielsen & Knardahl, 2014; Tran & Lumley, 2019) samples. On the other hand, many adaptive coping strategies also exist, which involves engaging constructively with distress that can come from experiences of loneliness. For instance, coping strategies in the family of self-reliance or support-seeking often contribute to an individual’s ability to proactively feel more in-control through constructing coping resources for themselves (such as calling a friend or saying positive affirmations to oneself, for example)—a process that often results in a greater sense of control and confidence for the individual (Skinner et al., 2003). Studies have found that the use of adaptive coping strategies (e.g. engagement, planning, problem-solving) is associated with higher levels of well-being (Muniandy et al., 2021, 2022; Tran & Lumley, 2019). Despite the handful of studies on coping with loneliness in the general population, no study to the authors’ knowledge has examined how autistic people or people with NDDs cope with loneliness in adaptive or maladaptive ways.

Considering the social, cognitive, and emotional development that occurs from childhood through adulthood, as well as potential fluctuations in loneliness over time (Mund et al., 2020), coping strategies may shift in tandem. For example, although cross-sectional, one study found that non-autistic older children (approximately age 12) use a greater number of coping strategies and more frequently use coping strategies that align with behavioral distraction, cognitive restructuring, and helplessness and use behavior regulation less often than younger children (approximately age 8) (Besevegis & Galanaki, 2010). As such, longitudinal data are needed to tell us how coping with loneliness may vary across development in autism and other NDDs.

## Study aims and hypotheses

Increased attention to loneliness in autism and NDD research has highlighted loneliness as a common experience in these populations that can have a negative effect on well-being and quality of life (Hymas et al., 2022; Umagami et al., 2022). Although cross-sectional studies have provided important insight into the pervasiveness and potential uniqueness of loneliness for autistic people or people with NDDs, longitudinal patterns of loneliness and how people cope with loneliness are understudied. Such longitudinal designs are needed to answer questions such as “are certain developmental stages especially vulnerable to loneliness?,” and “are those who are lonely as children likely to stay lonely over time?.” Working to address these questions could identify routes to promote well-being and improve quality of life across the lifespan for autistic people and people with NDDs. Therefore, in a longitudinal sample of autistic youth and youth with NDDs from approximately age 9 to their mid-20s, this study aimed to characterize continuity or changes in (1) experiences of loneliness, (2) perceptions of other people’s loneliness, and (3) strategies used to cope with loneliness across three developmental stages (childhood, adolescence, and early adulthood). We hypothesized that there would be developmental changes in participants’ own loneliness and perceptions of others’ loneliness such that average levels of loneliness would increase over time, and we would see rank-order stability across consecutive time points. We also hypothesized that the most common types of coping strategies would also change over time; lack of prior research precludes hypotheses specific to particular families of coping.

## Method

### Participants

A sample of 114 participants was drawn from a larger ongoing longitudinal study on autistic people and persons with other NDDs that began in the early 1990s and has followed participants for over 30 years. The subsample for the present study was selected based on completion of relevant measures which were collected during three developmental time frames, including childhood, adolescence, and young adulthood. Sample size varied across time and measure due to missing data (e.g. not administered the relevant measure, unable to complete self-report measure, did not return questionnaire) and therefore sample sizes and ages are described below for each relevant measure. Participants were originally consecutive referrals to three autism program sites (North Carolina, Illinois, and Michigan), though not all received autism diagnoses. Children recruited from North Carolina and Illinois were under age 3 at the start of the study; children recruited from Michigan joined the study when they were approximately 9 years old. Despite early developmental delays, 31.0% of the current sample never received a formal diagnosis of autism; these participants were retained in our analyses due to similar adult outcomes (e.g. work, friends, living situation) and characteristics (e.g. quality of life, mental health diagnoses) compared to the participants who received autism diagnoses (Lord et al., 2020; McCauley et al., 2020); retaining all participants also allows comparisons between autistic and non-autistic participants and increases statistical power. Diagnoses in the NDD group included multiple conditions such as intellectual disability, attention-deficit hyperactivity disorder, and learning disorder. Demographic information for the present subsample is presented in Table 1. The sample is predominantly White (80.7%) and male (79.8%) with an average verbal IQ of 90.27 (24.50), and about half reported a caregiver education of a college degree or more (46.3%). Compared to those lost due to attrition or missing data on all variables of interest (*N* = 140), the present sample (*N* = 114) had a significantly lower proportion of autistic participants (χ^2^ (1, *N* = 253) = 11.20, *p* < 0.05), participants of color (χ^2^ (1, *N* = 253) = 10.87, *p* < 0.05), higher IQs (*t*(224.95) = −6.51, *p* < 0.05), and lower autism symptom severity on an observational measure (i.e. Autism Diagnostic Observation Schedule (ADOS), which is described in “Measures” section) (*t*(251) = 19.43, *p* < 0.05), but did not differ by gender or caregiver education (*p* > 0.05).

**Table 1.** Sample demographic characteristics.

	M (SD)/*N* (%)
Race	
Person of color	20 (17.5)
White	92 (80.7)
Sex	
Male	91 (79.8)
Female	22 (19.3)
Caregiver education	
High school diploma equivalent or less	25 (21.9)
Some college or associate’s degree	34 (29.8)
4-year college degree	25 (21.9)
Graduate or professional degree	29 (25.4)
Diagnosis	
Autism	77 (67.5)
Other NDD	36 (31.6)
Recruitment site	
Illinois	35 (30.7)
North Carolina	47 (41.2)
Michigan	31 (27.2)
Cognitive verbal ability	
VIQ	90.27 (24.50)
Autism features	
ADOS CSS	4.64 (2.92)

NDD: neurodevelopmental disabilities; VIQ: Verbal Intelligence Quotient from standardized measure; ADOS CSS: Autism Diagnostic Observation Schedule Calibrated Severity Score.

### Procedure

A set of diagnostic and psychosocial instruments were collected through in-person visits, phone interviews, and questionnaire data from the same group of participants at multiple time points throughout their lives. In-person assessments and questionnaire data collection followed a longitudinal design and occurred multiple times from childhood through young adulthood (see “Measures” section for specific ages and time points). Postdoctoral fellows or licensed clinicians conducted the in-person assessments. Administrators were research reliable in the relevant measures and masked to participants’ previous assessment results. Diagnoses of autism or other conditions were made by the research team and presented to a panel of experienced clinicians who made consensus clinical diagnoses of autism and other conditions. All assessments were provided free of charge and included feedback on testing results. The larger longitudinal study was approved by Institutional Review Boards at various universities throughout the duration of the study. Caregivers, and when applicable, participants, provided written consent or assent prior to participating in the study.

### Measures

#### Autism Diagnostic Observation Schedule

The ADOS (Generic and second edition; Lord et al., 2000, 2012) was administered at each in-person visit by a clinician masked to previous diagnostic classification. The ADOS is a semi-structured observational tool designed to assess behaviors that are characteristic of autism (e.g. social communication, repetitive behaviors, and intense interests). The ADOS has multiple modules that are designed for different developmental and language levels. The ADOS was used for two purposes in the present study: (1) diagnostic characterization and (2) loneliness (coding of loneliness level and coping strategies, described below). Only participants who received ADOS Modules 3 or 4, which are intended for verbally fluent children/young adolescents and older adolescents/adults, respectively, were included; these two Modules are the only versions of the ADOS that include the questions about loneliness (described below). A sample of 110 participants had Module 3 or 4 ADOS data from at least one time point; the full sample (*n* = 114) had received ADOSs for diagnostic purposes. The ADOS was completed at approximately age 9 (M = 9.45, SD = 1.16; *n* = 67), age 19 (M = 18.91, SD = 1.35; *n* = 76), and age 25 (M = 25.48, SD = 1.62; *n* = 62) and was available from 38 participants at 3 time points, 30 participants at 2 time points, and 42 participants at 1 time point.

##### Coding ADOS loneliness data

Based on ADOS protocol forms, responses to loneliness items were qualitatively coded for (1) level of loneliness, (2) loneliness-related factors, and (3) loneliness coping strategies. Specifics of each coding approach are described below. Reliability was calculated across coders and achieved 80% or greater agreement on the first 20% of data to ensure accurate understanding and application of codes. All data were double coded. Regular coding meetings were held to discuss coding questions or issues, and consensus was reached on all discrepant items. Sample sizes vary across different codes due to a variety of circumstances in which data were not available to be coded including: the examiner didn’t ask the question, the participant didn’t provide an answer to the item, or the examiner didn’t indicate the participant’s response on the protocol form.

##### Coding ADOS loneliness level and loneliness-related factors

Responses to the items “Do you ever feel lonely?” and “Do you think other (young) people in your circumstances [other people your age; ADOS-2] ever feel lonely?” were coded qualitatively using content analysis (Elo & Kyngäs, 2008). The procedure involved the following steps: (1) preparing for coding by becoming familiar with the data, (2) generating initial categories, (3) searching for hierarchical structure of categories and subcategories (only for loneliness-related factors), (4) revising and defining codes and categories, and (5) reporting results. Analyses of loneliness level and loneliness-related factors were conducted from an inductive approach (i.e. identifying codes directly from the data). Level of loneliness was ultimately coded on a 5-point scale indicating level of loneliness (with higher values reflecting more loneliness) yielding both ADOS Loneliness–Self Ratings and ADOS Loneliness–Others Ratings (see Supplemental Table 1). Spontaneous elaborations on the experience of loneliness were coded as Loneliness-Related Factors (see Supplemental Table 1).

##### Coding ADOS loneliness coping strategies

The ADOS item related to coping with loneliness (“Are there things that you do to help yourself feel better?”) was coded using the same system used in previous studies on coping with loneliness (Besevegis & Galanaki, 2010; Vasileiou et al., 2019), which is a deductive approach (i.e. applying an existing coding system to the data). That is, coping responses were classified within one of 12 *Families of Coping* (e.g. Accommodation), each of which aligns with one or more specific *Coping Strategy* (e.g. Distraction) (see Supplemental Table 2 for all 12 Families of Coping, specific Coping Strategies, and examples from this data). Consistent with previous research (Besevegis & Galanaki, 2010), a sum of the number of coping strategies was also calculated for each participant. In addition, for Behavioral Distraction, the specific distraction activity was coded, and for Instrumental Action, the mode of social contact and contact person was coded using the content analysis approach described above.

#### Asher Loneliness Scale

The Asher Loneliness Scale, also called the Illinois Loneliness and Social Dissatisfaction Questionnaire, is a self-reported measure of loneliness originally developed for use with children (Asher & Wheeler, 1985, adapted from Asher et al., 1984); the current version includes minor modifications for developmental appropriateness (e.g. “I’m lonely when I’m at school” was modified to “I’m lonely when I’m at school or work”). A subset of participants in the current study (*n* = 46) completed this measure at least once during the study. Based on a critical review of loneliness measures by the questionnaire’s author (Weeks & Asher, 2012), we retained only “pure” loneliness items (i.e. items that reference being or feeling lonely; 8 items) to avoid including confounding items that tap into hypothesized causes of loneliness (e.g. lack of friendships; Weeks & Asher, 2012). For example, “I’m lonely when I’m around other people” is a pure loneliness item, while items such as “It’s hard for me to make friends” or “I don’t get along with other people” taps into and is confounded by other social factors. Therefore, responses to pure loneliness items were summed; higher values indicated more loneliness. Response options for each item are on a 5-point Likert-type scale from not true (1) to always true (5). The Asher Loneliness Scale was completed multiple times from approximately age 17 through 24 (maximum of 5 times). As with many loneliness measures (see Umagami et al., 2022), this measure has not yet been psychometrically evaluated in samples of autistic people. To maximize available data for these analyses, questionnaire data were binned into three time points: ages up to and including 17 (M = 17.03, SD = 0.38; *n* = 25), ages 18–20 (M = 19.24, SD = 0.63; *n* = 36), and ages 21 and older (M = 22.62, SD = 0.93; *n* = 28).

#### IQ assessments

A set of standardized cognitive assessments was used to measure cognitive abilities at each in-person assessment. A developmentally appropriate assessment was selected from the following: Wechsler Abbreviated Scale of Intelligence (WASI; Wechsler, 1999), Wechsler Intelligence Scale for Children (WISC-III; Wechsler, 1991), and Differential Abilities Scale (DAS; Elliott et al., 1990, 2007). Ratio IQs were calculated from age equivalents when raw scores did not fall within standardized score ranges.

### Data analytic plan

First, preliminary analyses were run to determine whether participants with and without autism diagnoses differed on loneliness measures (i.e. ADOS Loneliness Ratings and Asher Loneliness Scale) using parametric (independent samples *t*-tests) and non-parametric tests (Mann–Whitney *U*), as appropriate. ADOS Loneliness–Self Ratings were then correlated with questionnaire data (i.e. Asher Loneliness Scale) using Kendall’s tau-b, given the ordinal nature of the ADOS Loneliness Ratings. Second, in line with Aims 1 and 2, to examine mean/median level change in loneliness across each time point, we used paired-samples *t* tests and Wilcoxon signed-rank tests for the Asher Loneliness Scale scores and ADOS Loneliness Ratings, respectively. To further characterize ADOS Loneliness Ratings, we examined frequencies and percentages of each Loneliness Rating at each time point. Third, in line with Aims 1 and 2, to examine rank-order stability in loneliness over time, Pearson correlations and Kendall’s tau-b were conducted with the Asher Loneliness Scale scores and ADOS Loneliness Ratings, respectively. Fourth, as an exploratory analysis, we compared ADOS Loneliness Ratings for Self versus Others using Wilcoxon signed-rank test. Fifth, in line with Aims 1 and 2, frequencies and percentages of Loneliness-Related Factors were calculated at each time point. Sixth, to address Aim 3, frequencies and percentages of each Loneliness Coping Strategy category were calculated at each time point. Number of Loneliness Coping Strategies was also calculated, consistent with previous research (Besevegis & Galanaki, 2010) and correlated with ADOS Loneliness Ratings for Self to determine whether having more “coping tools” is advantageous. As an exploratory analysis, differences in ADOS Loneliness–Self Ratings were examined between those who endorsed versus did not endorse use of certain coping strategies using Mann–Whitney *U* tests. For analyses conducted within time point, analyses were re-run excluding participants who only have one assessment; interpretation of results did not change.

### Community involvement

These data are pulled from a larger longitudinal study that has been ongoing for over 30 years in which there has been community involvement throughout the duration of the study. In particular, participants and their families are regularly (approximately every 6 months) contacted by phone by research assistants, graduate students, and post doctoral scholars to ask for their opinions on important areas of research focus for the longitudinal study. This study also has a formal advisory board that meets once a year facilitated by the study’s PI and additional research team members to provide input and feedback on the degree to which the study is meeting their goals and the research team’s goals. Multiple authors on this paper are clinicians who actively see autistic children and adults in their clinical practice. None of the authors identify as autistic or neurodivergent.

## Results

### Preliminary analyses

Autistic and non-autistic participants did not differ on any of the ADOS Loneliness Ratings for self or for others (*p* > 0.05). Two out of the three time points of the Asher Loneliness Scale also did not differ between those with and without autism. Only at age 17, autistic participants (M = 17.72, SD = 7.65, *n* = 18) had higher Asher Loneliness Scale scores on average compared to the participants with NDDs (M = 10.45, SD = 3.14, *n* = 7) (*t*(23) = 2.49, *p* = 0.02). Therefore, all participants are retained in subsequent analyses.

Correlations between ADOS Loneliness–Self Ratings and Asher Loneliness Scale scores were generally positive, particularly for measurement occasions close in time (Asher Loneliness Age 19 and ADOS Loneliness Age 19: *τ*_b_ = 0.40, *p* = 0.01, *n* = 28; Asher Loneliness Age 23 and ADOS Loneliness Age 25: *τ*_b_ = 0.44, *p* = 0.01, *n* = 22). See Table 2 for full correlation matrix and descriptive statistics.

**Table 2.** Descriptives and correlations of Asher loneliness scores and ADOS loneliness ratings.

Variable	*n*	M	SD	1	2	3	4	5	6	7	8
1. Asher Loneliness—Age 17	25	15.69	7.42	–							
2. Asher Loneliness—Age 19	36	15.48	5.75	0.60**	–						
3. Asher Loneliness—Age 23	28	17.68	7.22	0.49	0.66**	–					
4. ADOS Loneliness Self—Age 9	57	3.00	–	0.14	0.40*	0.15	–				
5. ADOS Loneliness Self—Age 19	68	3.00	–	0.02	0.40*	0.19	0.40*	–			
6. ADOS Loneliness Self—Age 25	58	3.00	–	0.29	0.37*	0.44*	0.40*	0.42**	–		
7. ADOS Loneliness Others—Age 9	29	4.00	–	–	–	–	0.46**	–0.39	0.34	–	
8. ADOS Loneliness Others—Age 19	59	3.00	–	–0.13	–0.23	–0.18	0.15	0.21*	0.02	–	
9. ADOS Loneliness Others—Age 25	56	4.00	–	–0.01	0.33*	0.14	0.05	0.28*	0.39**	0.27	0.22

ADOS: Autism Diagnostic Observation Schedule.

T1: **p < 0.01; *p < 0.05; ^*p* < 0.10; values not reported when cell size was smaller than *n* = 10; M = mean for Asher Loneliness and median for ADOS Loneliness.

### Loneliness over time

#### Mean/median change

ADOS Loneliness Ratings for Self and Others are presented in Table 3. Across the three time points, Age 25 had the lowest proportion of participants endorsing no loneliness (15.52%) and the highest proportion endorsing loneliness in the affirmative (i.e. yes or definitely yes; 34.49%). ADOS Loneliness–Self Ratings did not change significantly from age 9 to 19 (*z* = −1.52, *W* = 34.00, SE = 17.10, *p* = 0.13, *n* = 27), but increased significantly from age 19 to 25 (*z* = 2.11, *W* = 159.50, SE = 25.89, *p* = 0.04, *n* = 43) (see Supplemental Table 3). In contrast, across a shorter span of time, Asher Loneliness scores did not significantly change from age 17 to 19 *t*(19) = 0.45, *p* = 0.65 or from age 19 to 23 *t*(21) = −1.10, *p* = 0.29. Given the lower Asher Loneliness scores of non-autistic participants at age 17 (described above in “Preliminary analyses” section), comparisons of Asher Loneliness scores from age 17 to 19 were re-run excluding participants without autism, and results remained identical.

**Table 3.** Loneliness ratings on ADOS.

	Age 9		Age 19		Age 25	
	*n*	%	*n*	%	*n*	%
Loneliness–Self	(*n* = 57)		(*n* = 68)		(*n* = 58)	
No (1)	15	26.32	19	27.94	9	15.52
Not really/Not often/Rarely (2)	1	1.75	12	17.65	9	15.52
Sometimes/At times/Once in a while/Probably (3)	24	42.11	26	38.24	20	34.48
Yes/Yeah/Sure/I’m sure they do (4)	14	24.56	11	16.18	16	27.59
Yes, definitely/All the time/A lot (5)	3	5.26	0	0.00	4	6.90
Loneliness–Others	(*n* = 29)		(*n* = 59)		(*n* = 56)	
No (1)	10	34.48	9	15.25	5	8.93
Not really/Not often/Rarely (2)	0	0.00	4	6.78	2	3.57
Sometimes/At times/Once in a while/Probably (3)	4	13.79	18	30.51	6	10.71
Yes/Yeah/Sure/I’m sure they do (4)	13	44.83	24	40.68	34	60.71
Yes, definitely/All the time/A lot (5)	2	6.90	4	6.78	9	16.07

ADOS: Autism Diagnostic Observation Schedule.

Percentages are based on valid data (excluding missing or uncodable responses) as reflected in reported sample size for each age.

For the ADOS Loneliness–Others Ratings, similar to ratings for Self, Age 25 had the lowest proportion of participants endorsing no loneliness (8.93%) and the largest proportion endorsing loneliness (yes or definitely yes; 76.78%) In addition, there was a significant increase in ratings from age 19 to 25 (*z* = 2.65, *W* = 205.00, SE = 29.60, *p* = 0.01, *n* = 34; see Supplemental Table 3); sample size from age 9 to 19 was too small to make meaningful comparisons.

#### Correlations

ADOS Loneliness–Self Ratings were significantly correlated over time from age 9 to 19 (*τ*_b_ = 0.40, *p* = 0.02, *n* = 27) and 19 to 25 (*τ*_b_ = 0.41, *p* = 0.001, *n* = 43), as well as from age 9 to 25 (*τ*_b_ = 0.40, *p* = 0.01, *n* = 29). Similarly, Asher Loneliness Scores were positively and significantly correlated over time from age 17 to 19 (*r*(19) = 0.60, *p* = 0.005) and 19 to 23 (*r*(21) = 0.66, *p* < 0.001). The correlation of the Asher Loneliness Scores from age 17 to 23 was positive but not significant (*r*(10) = 0.49, *p* = 0.13) likely due to small sample size. ADOS Loneliness–Others Ratings were not significantly associated over time.

### ADOS Self versus Others loneliness ratings

There were significant differences between ADOS Loneliness Ratings of Self versus Others at ages 19 (*z* = 3.37, *W* = 491.00, SE = 57.46, *p* < 0.001, *n* = 55) and 25 (*z* = 3.75, *W* = 512.50, SE = 57.28, *p* < 0.001, *n* = 54) such that the sample rated others as lonelier than themselves (Supplemental Table 4). There was no difference in ADOS Loneliness Ratings of Self versus Others at age 9 (*z* = 1.40, *W* = 94.50, SE = 18.90, *p* = 0.16, *n* = 28).

### Loneliness-related factors

**Figure 1.**
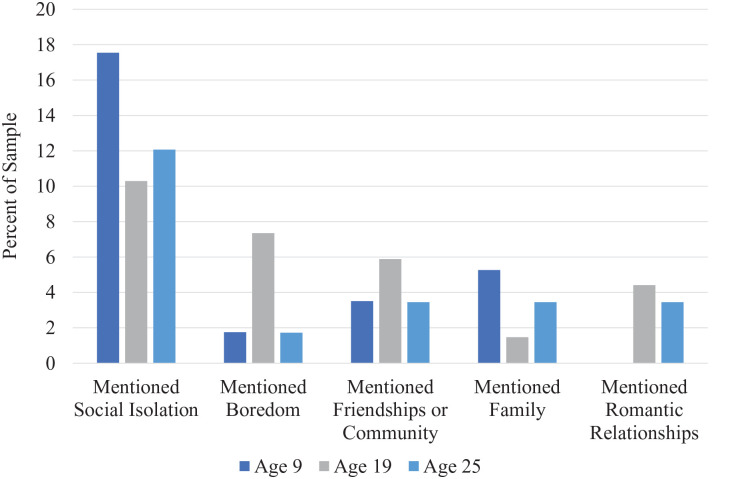
Percent endorsement of loneliness-related factors—Self.

**Figure 2.**
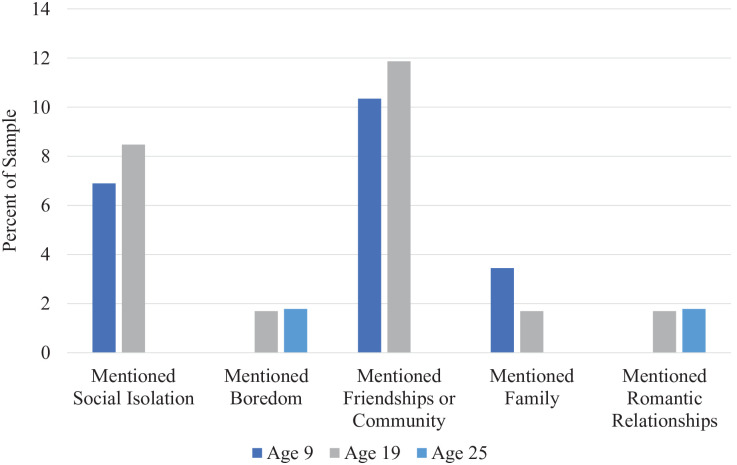
Percent endorsement of loneliness-related factors—Others.

In addition to describing their level of loneliness during the ADOS, participants spontaneously provided information about their experiences of loneliness including identifying certain types of relationships (e.g. friends) and contextual factors (e.g. boredom), among other details. For the sake of parsimony, the five most common loneliness-related factors related to themselves and others are presented in Figures 1 and 2, respectively. See Supplemental Table 5 for details regarding endorsement of all categories and subcategories of loneliness-related factors.

When describing their own loneliness, social isolation (i.e. being alone) was the most frequently mentioned factor related to loneliness, yet this was mentioned less often at ages 19 and 25 than age 9. Boredom was mentioned as a contributing factor to loneliness, particularly at age 19 (e.g. “When I’m bored, like yesterday [I had] nothing to do [except] play with some stuff, [and watch] TV, a lot of TV”). Romantic relationships were only mentioned at ages 19 and 25.

When describing other people’s experience of loneliness, friendships were the most commonly mentioned factor, especially at ages 9 and 19. Having friendships was often mentioned as a protective factor against loneliness (e.g. “Not really, has friends and family who care,” “many don’t seem lonely and have lots of friends”), while the lack of friendship is a risk factor (e.g. “sometimes, because they don’t have many friends,” “some do because it’s hard to make friends”). Compared to descriptions of their own loneliness, friendships were more commonly mentioned and social isolation was less often mentioned in relation to other people’s loneliness. Boredom was mentioned infrequently when describing other people’s loneliness. Similar to descriptions of their own loneliness, romantic relationships were only mentioned at ages 19 and 25.

### Coping with loneliness

Across all three time points, the most common coping strategy identified on the ADOS in response to loneliness was Behavioral Distraction (a type of Accommodation) followed by Instrumental Action (a type of Problem-Solving) (Table 4). Use of Behavioral Distraction increased from age 9 (approximately 39%) to ages 19 and 25 (over 50%). The proportion of the sample using Instrumental Action also increased from ages 9 and 19 (approximately 30%) to age 25 (43%). Use of Passivity (i.e. doing nothing) was highest at age 9 (25%). Avoidance Behaviors (e.g. drinking alcohol) was highest at age 25 (12%). Overall, participants reported using a greater number of coping strategies as they got older; 43% of the sample reported using two or more coping strategies at age 25, compared to 30% at age 19 and only 21% at age 9. Number of coping strategies was positively correlated with ADOS Loneliness–Self Ratings at age 19 (*τ*_b_ = 0.29, *p* = 0.007, *n* = 68) and 25 (*τ*_b_ = 0.41, *p* < 0.001, *n* = 59), but not at age 9 (*τ*_b_ = −0.04, *p* = 0.77, *n* = 58).

**Table 4.** Coping strategies for loneliness.

Family of coping	Category	Age 9		Age 19		Age 25	
		(*n* = 28)		(*n* = 50)		(*n* = 49)	
		*n*	%	*n*	%	*n*	%
Problem-solving	Instrumental action	8	28.57	15	30.00	21	42.86
	Self-improvement	0	0.00	1	2.00	0	0.00
	Cognitive problem-solving	1	3.57	1	2.00	1	2.04
	Strategizing/planning	1	3.57	1	2.00	1	2.04
Helplessness	Passivity	7	25.00	1	2.00	3	6.12
Escape	Cognitive avoidance	1	3.57	1	2.00	0	0.00
	Avoidant behaviors	0	0.00	2	4.00	6	12.24
	Denial	0	0.00	0	0.00	1	2.04
	Wishful thinking	0	0.00	1	2.00	0	0.00
Self-reliance	Emotion regulation	2	7.14	5	10.00	4	8.16
	Behavior regulation	1	3.57	0	0.00	1	2.04
	Emotional expression	0	0.00	0	0.00	1	2.04
	Emotion approach	0	0.00	0	0.00	3	6.12
Support seeking	Instrumental aid	0	0.00	0	0.00	1	2.04
	Spiritual support	2	7.14	3	6.00	1	2.04
Accommodation	Behavioral distraction	11	39.29	30	60.00	25	51.02
	Acceptance	0	0.00	1	2.00	2	4.08
Submission	Behavioral submission	0	0.00	1	2.00	0	0.00
Delegation	Complaining/whining	0	0.00	1	2.00	0	0.00
Social isolation	Concealment	0	0.00	1	2.00	0	0.00
	Avoiding others/withdrawal	0	0.00	1	2.00	1	2.04

Percentages are based on valid data only (excluding missing or uncodable responses) as reflected in reported sample size for each time point.

Behavioral Distraction strategies included a range of activities (Table 5). In this sample, of those who endorsed using Behavioral Distraction, the use of non-electronic activities was highest at age 9 (100%), while the use of electronic activities was highest at ages 19 and 25 (approximately 57%–61%). Vague responses (i.e. keep myself busy) were more common over time (i.e. at older ages). Those who endorsed use of Behavioral Distraction had higher ADOS Loneliness–Self Ratings at ages 19 (*U* = 257.50, *p* < 0.001) and 25 (*U* = 299.50, *p* = 0.003), but not at age 9.

**Table 5.** Behavioral distraction and social contact strategies used to cope with loneliness.

Strategies	Age 9		Age 19		Age 25	
	*n*	%	*n*	%	*n*	%
Behavioral distraction strategies	*n* = 11		*n* = 30		*n* = 25	
Electronic activities (e.g. video games, Internet, TV, movies)	1	9.09	17	56.67	16	64.00
Non-electronic activities (e.g. books, homework, board games, sports)	11	100.00	13	43.33	2	8.00
Vague (e.g. keep busy/distracted)	0	0.00	9	30.00	5	20.00
Listen to music	0	0.00	5	16.67	6	24.00
Nap/sleep	2	18.18	1	3.33	2	78.00
Eating/drinking	0	0.00	3	10.00	3	12.00
Social contact strategies	*n* = 8		*n* = 15		*n* = 21	
Contact person						
Family (parent, sibling, relative)	3	37.50	3	20.00	5	23.81
Friend	3	37.50	7	46.67	5	23.81
Significant other/romantic prospects	0	0.00	1	6.67	2	9.53
Unclear	3	37.50	5	33.33	11	52.38
Mode of contact						
Phone (call or text)	0	0.00	3	20.00	6	28.57
In-person	6	75.00	4	26.67	10	47.62
Social media	0	0.00	1	6.67	3	14.29
Unclear	2	25.00	8	53.33	6	28.57

*Note.* Percentages are based on the number of participants who endorsed “Behavioral Distraction” or “Instrumental Action” as a coping strategy at that time point, as reflected in reported sample size for each time point. Strategies are not mutually exclusive.

Of those who endorsed using Instrumental Action (i.e. seeking social contact), a fairly even proportion of the sample identified a family member, friend, or no specific person (i.e. contact someone) (Table 5). Although few, more people indicated reaching out to potential romantic interests in adulthood than earlier in the study. Mode of contact shifted from primarily in-person at age 9 to a combination of in-person and phone contact at ages 19 and 25. No differences in ADOS Loneliness–Self Ratings emerged based on the use of Instrumental Action coping strategy.

## Discussion

Building on the growing body of research indicating that loneliness is common and associated with quality of life and well-being among autistic people and people with other NDDs (Alexandra et al., 2018; Hymas et al., 2022; Umagami et al., 2022), the current study longitudinally investigated loneliness and related coping strategies from childhood through adulthood in autistic people and people with non-spectrum NDDs. As people in our sample aged, they reported higher levels of loneliness and use of more strategies to cope with loneliness over time during the interview section of the ADOS. Notably, many, but not all, participants reported that they felt lonely; within each developmental stage, approximately a third of the sample reported never or rarely feeling lonely. Consistent with research among non-autistic people, common loneliness coping strategies were characterized by social reconnection or avoidance. Overall, findings highlight adulthood as a particularly vulnerable time for loneliness among autistic people and people with NDDs.

Although there were no changes in loneliness from childhood to late adolescence, loneliness was found to increase from adolescence through early adulthood based on responses on the ADOS. Although a direct comparison cannot be made, the pattern observed in the current work appears to be relatively distinct from that identified in the general population in which loneliness is stable during adolescence and adulthood overall in the general population (Mund et al., 2020) and somewhat similar to a subset of the general population in which loneliness remains chronically high or increases from childhood into adulthood (van Dulmen & Goossens, 2013). This increase in loneliness in our sample may be attributed to fewer and less structured social opportunities in the lives of autistic young adults. For example, many transition out of high school and into contexts with greater potential for feelings of isolation, such as navigating employment or aging out of the social services and supports typically offered to adolescents (Orsmond et al., 2004; Tobin et al., 2014). In addition, a decrease in teacher and parent-mediated socialization (e.g. recess and lunch groups, team assignments) may also contribute to greater reports of loneliness as individuals adjust to more independent and self-directed socialization styles. A final possible explanation could be due to maturation; perhaps adults are simply more attuned to their own loneliness or are more comfortable with openly discussing it than when they were children. In contrast with findings from the ADOS, the stability in loneliness based on the Asher Loneliness Scale may be due to the relatively small sample size available for this measure, the shorter time span between measurements, as well as difference in terms of method of reporting (i.e. questionnaire vs interview). Perhaps, people, particularly adults who likely have more social awareness than children, are more comfortable reporting loneliness on a questionnaire as opposed to verbally to an interviewer. Nonetheless, these findings add to a growing call for social supports aimed at helping autistic adults who wish to do so navigate interpersonal relationships and form social connections.

Evidence for rank-order stability (i.e. within-person correlations over time) was also found by the current study. Based on questionnaire data and ADOS interview questions gathered across three developmental stages (childhood, late adolescence, and emerging adulthood), individuals who reported higher loneliness earlier in life were likely to maintain their reports of higher loneliness overtime, relative to others in the sample. Research in the general population identifies persistent negative cognitive biases in lonelier individuals that may propagate a sense of rejection expectation (Spithoven et al., 2017). That is, lonelier individuals may feel a sense of futility with respect to changing their loneliness, in turn, leading to behaviors that sustain their loneliness.

The current sample of autistic individuals perceived other people to be lonelier than themselves. This finding is in contrast with studies in the general population in which people tend to report harsher self-perceptions of loneliness compared to perceptions of other people’s loneliness (Cacioppo & Hawkley, 2005; Duck et al., 1994; Haslam, 2022; Spithoven et al., 2017; Vanhalst et al., 2013, 2015). Because of social communication differences inherent to autism, autistic people may be less vocal about the degree of their own loneliness relative to others’. Difficulties identifying and describing internal emotional experiences, called alexithymia, that commonly affect people on the spectrum (Kinnaird et al., 2019), in addition to potential differences in self-awareness that may increase with age (Huggins et al., 2020), may also contribute to under-reporting of one’s own loneliness. Furthermore, participants may view contributors to other people’s experiences of loneliness differently than their own. For example, participants identified friends more often when describing other people’s experience of loneliness compared to their own.

Notably, other than the questionnaire measure of loneliness at age 17, our findings revealed highly similar levels of loneliness for autistic and non-autistic NDD participants in our sample. These similarities across groups parallel other findings showing similar trajectories and outcomes for individuals with NDD and autistic individuals (Lord et al., 2020; McCauley et al., 2020) and their family members (Schiltz et al., 2023; Singer et al., 2023). Research should continue to identify unique and disparate experiences, challenges, and strengths of autistic people and people with non-spectrum developmental delays.

Several factors pertaining to experiences of loneliness in autistic people were found to vary across developmental stages. Social isolation was mentioned in relation to loneliness, but more so in childhood than later in life. Given that loneliness is a negative emotional experience that comes from the *perception* of a social deficit, children may be more sensitive to loneliness as a result of physically being alone, while adults may be more likely to use time alone to garner restoration and social “re-charging” (Elmose, 2020). Furthermore, considering that boredom emerged as a factor related to loneliness, it is important for individuals to feel busy with daily tasks and responsibilities. This is particularly important for adults, given the potential for unstructured free time after exiting secondary school and high underemployment rates among autistic adults (Hedley et al., 2017). When integrated into self-directed visual schedules, leisure activities have been found to not only counter feelings of boredom that may arise in the face of otherwise unstructured time but also increase a sense of independence and autonomy (Nepo et al., 2020). Furthermore, deliberate leisure and recreation time among autistic people has been found to bolster stress management and attenuate perceived life stress (Bishop-Fitzpatrick et al., 2017), contribute to improved quality of life outcomes (Bishop-Fitzpatrick et al., 2017; Stacey et al., 2018), and reduce depression (Stacey et al., 2018), suggesting positive far-reaching effects of structured leisure activities for autistic people.

In the current sample, the types of relationships mentioned alongside loneliness varied within and across developmental stages. For example, friends tended to be mentioned more than family in relation to loneliness, especially when describing other’s loneliness. Perhaps the voluntary choice involved in reciprocal friendships holds more weight than the potentially obligatory nature of family relationships. In addition, although infrequent, romantic relationships were mentioned more often by adults than children and adolescents, which is consistent with normative developmental changes. This finding is in alignment with prior research indicating that autistic adults identify romantic relationships as a lower priority than other factors such as friendships, education, and employment (Gotham et al., 2015). Accordingly, these results highlight loneliness as a subjective, multifaceted, and dynamic experience among autistic people.

Analyses of coping strategies used to manage feelings of loneliness revealed similar patterns to those identified by previous research among non-autistic children and adults (Besevegis & Galanaki, 2010; Vasileiou et al., 2019), as well as a recent doctoral thesis (Umagami, 2023). In particular, the two most common coping strategies identified in our sample were use of distractions (e.g. watching TV) and seeking social contact (e.g. calling a friend), which are types of accommodation and problem-solving coping, respectively. Regarding changes in coping strategies over time, the number of different coping strategies, as well as use of behavioral distraction in particular, increased with age. Adults may be more flexible with the type of coping strategy they use, meaning they have more “tools” to use to cope with loneliness. The use of electronic and non-electronic distraction strategies increased and decreased, respectively, with age. These changes may be related to increased availability, access, and popularity of technology for this particular cohort (who was recruited in the early 1990s).

Examining the links between level of loneliness and coping strategy use revealed that while those who either use behavioral distraction or use a greater number of coping strategies report *higher* concurrent levels of loneliness, use of instrumental action was unrelated to level of loneliness. In the general population, use of distractions is considered maladaptive, while seeking social contact is considered adaptive, as these coping responses further jeopardize or constructively uplift the individual seeking relief from loneliness, respectively (Skinner et al., 2003). Given the typically “adaptive” nature of instrumental action, the lack of a difference in loneliness between those who use and do not use instrumental action (i.e. seek social contact) was somewhat surprising. However, it is important to remember that experiences of loneliness result from perceptions (i.e. cognitions), which may or may not be directly tied to behavioral change (i.e. being with other people). In addition, social initiation does not necessarily imply acceptance; people may be rejected or ignored. It may also be that the effect of seeking social contact on loneliness emerges over time, rather than concurrently. Regarding the link between greater loneliness and use of distraction coping, given that trying to distract one’s self doesn’t address the core issue causing the distress, but instead masks unpleasant emotions temporarily and is considered maladaptive, feelings of loneliness likely persist once the distractor is removed. The link between use of more coping strategies and higher levels of loneliness was not altogether unsurprising; those who are not lonely do not need to cope. The need to use a great number of coping strategies may also be due to potential ineffectiveness of certain coping strategies. That is, there may be a need to try different strategies when one is not working; the effectiveness of coping strategies is often tied to the particulars of the stressor including temporal deployment (e.g. before, during, or after the peak of feeling lonely) and social context (e.g. loneliness due to peer rejection vs feeling disconnected with family members) (Compas et al., 2017).

The findings from the current study have implications for clinical practice, policy, and future research among autistic people and those with NDDs. In order to address the potential for increased loneliness into adulthood, clinicians working with autistic people should assess whether the person’s current social needs are being met, and if not, help foster and maintain social connection. This may involve helping identify opportunities for social interaction that feel reasonable to them. Ideally, this would occur well before adulthood in attempts to provide a foundation for social success later in life, with ongoing support to help navigate the changing milieu of adulthood. Moreover, clinicians should aim to identify and intervene on loneliness early in development, given the longitudinal persistence of loneliness over time revealed by the current study. From a larger societal and policy perspective, additional supports and structure from governmental agencies during transition ages may help to bridge the social gap between adolescence and adulthood. Because perceived social acceptance has been found to attenuate feelings of loneliness (Vanhalst et al., 2013), it may also be beneficial to continue increasing awareness and knowledge of autism in society more broadly, consistent with priorities identified by the autistic community (Gotham et al., 2015) and recommendations by other studies (Umagami, 2023).

Despite the many strengths of this study such as the longitudinal study design, findings from this research study should be considered within the scope of the following limitations. First, although the overall sample was sizable, the number of participants in the current sample with consecutive measurements was limited, and there was sparseness for some of the qualitative codes, particularly for Loneliness-Related Factors and Coping. Varying sample size across time points for qualitative data may have impacted findings. As such, study results, particularly across time, should be interpreted with caution and replicated with a larger sample. Given the specificity of this cohort (i.e. toddlers recruited and diagnosed in the early 1990s), the generalizability of these findings to other clinical samples diagnosed later in development or time is unknown. Another limitation of the current work pertains to our use of participants’ responses on the ADOS as a metric of loneliness (e.g. asking if participants “ever” feel lonely). Possibilities of misinterpretation and influence of other factors (e.g. personality factors, comfort verbally discussing loneliness, shyness, social behavior) remain, despite evidence of validity found via correlations between ADOS Loneliness Ratings and Asher questionnaire responses, albeit moderate in strength. In addition, the Asher questionnaire does not have norms available to help contextualize the current level of loneliness nor has this measure been validated for use among autistic people. Furthermore, causality cannot be inferred from these results (i.e. coping strategy use and level of loneliness). Accordingly, future studies should aim to recruit larger samples that will allow examination of directionality of effects across time (e.g. cross-lagged panel analyses). Finally, given that approximately a third of the sample at each time point reported low levels of loneliness during the ADOS interview, future work should aim to better understand factors contributing to resilience in this subgroup.

Overall, this longitudinal study highlights the varied experiences of loneliness in autism and other NDDs and provides evidence that feelings of loneliness do not seem to remit, and may actually increase, across development. As such, there is a need for greater support of autistic individuals in navigating the transition to the social world of adulthood.

## Supplemental Material

sj-docx-1-aut-10.1177_13623613231217337 – Supplemental material for A longitudinal study of loneliness in autism and other neurodevelopmental disabilities: Coping with loneliness from childhood through adulthood Supplemental material, sj-docx-1-aut-10.1177_13623613231217337 for A longitudinal study of loneliness in autism and other neurodevelopmental disabilities: Coping with loneliness from childhood through adulthood by Hillary Schiltz, Dena Gohari, Jamie Park and Catherine Lord in Autism
