# Supplementary material for: A longitudinal study of loneliness in autism and other neurodevelopmental disabilities: Coping with loneliness from childhood through adulthood
Source: Autism. 2023 Dec 28;28(6):1471–86. doi: 10.1177/13623613231217337 (PMC11132953; doi:10.1177/13623613231217337)
Supplement: sj-docx-1-aut-10.1177_13623613231217337 – Supplemental material for A longitudinal study of loneliness in autism and other neurodevelopmental disabilities: Coping with loneliness from childhood through adulthood [file sj-docx-1-aut-10.1177_13623613231217337.docx]

| **Supplemental Table 1** | |  |
| --- | --- | --- |
| *Content Coding of ADOS Loneliness Item* | |  |
| **ADOS Loneliness Level Ratings (Self & Others)** | | |
| Rating | Description | |
| 1 | No | |
| 2 | Not really/Not often/Rarely/Seldom/Not much/little bit | |
| 3 | Sometimes/At times/Every now and then/Not all the time/Once in a while/Maybe | |
| 4 | Yes/Yeah/Sure/I'm sure they do/Probably/I think so | |
| 5 | Yes, definitely/All the time/A lot/Yes, of course | |
| 777 | IDK/Not Sure | |
| 888 | Not Codable | |
| 999 | Missing / No Answer | |
|  | | |
| **ADOS Loneliness Related Factors** | | |
| Category | Subcategory & Description | |
| Social or Emotional Loneliness | Social Isolation: Mentioned social isolation "being alone" | |
|  | Emotional Loneliness: Mentioned emotional experience of loneliness (e.g., feeling upset, discontent, sad about not having friends) | |
| Contextual Factors | Boredom: Mentioned boredom or not having enough to do | |
|  | Days/Seasons: Mentioned certain days or seasons or situations | |
| Types of Relationships | Friends Mentioned friendships or a group of people or community (either lack or or presence of) | |
|  | Family: Mentioned family members (either lack or or presence of) | |
|  | Romantic: Mentioned romantic relationships (either lack or or presence of) | |
| Individual Factors | Characteristics: Mentioned personal characteristic or disposition (e.g., introversion) | |
| Loneliness Changes | Change: Mentioned changes in loneliness or experience of loneliness (e.g., I get used to it) | |
|  | Difference: Mentioned difference between others and self in terms of loneliness | |

| **Supplemental Table 2**  *12 Families of Coping Strategies with Examples* | | |
| --- | --- | --- |
| ***Family of Coping*** | *Category* | *Example* |
| **Problem Solving** | Instrumental Action | Text significant other or friend  Go out with people with similar interests  Call my mom on the phone  Text friend, ask how they're doing  Go to my friend's house and play with them  Just find a friend in my school  Meet people on apps/going out  Force yourself to be around more people |
|  | Self-improvement | Practice techniques |
|  | Cognitive problem solving | Think deeply |
|  | Strategizing/Planning | Working on figuring out |
| **Helplessness** | Confusion | Wondering why they felt lonely* |
|  | Passivity | Wait for it to pass |
| **Escape** | Cognitive Avoidance | I just forget about it  Take mind off  I just don’t think about it |
|  | Avoidant Behaviors | Dives into dirty things, sext  Drink alcohol  Smoke pot |
|  | Denial | Don’t talk about issues |
|  | Wishful Thinking | Fantasize…about girls |
|  | Behavioral Submission | Cut themselves |
| **Self-Reliance** | Emotion Regulation | Just relax  Be happy  Doing something that makes you feel good |
|  | Behavior Regulation | I can try to control myself |
|  | Emotional Expression | Crying* |
|  | Emotion Approach | If need to stay sad, stay sad |
| **Support/Contact Seeking** | Instrumental Aid | Proper channels, see someone |
|  | Spiritual Support | God help me  Pray a prayer  Try to please God  Ask God for guidance |
| **Accommodation** | Behavioral Distraction | Watch TV and movies, play, social media, read, music, internet, look up good looking guys, focus on something else, distractions, draw pictures, eat, dive into hobbies, puzzle, make some pancakes, work out, do schoolwork |
|  | Minimization | Telling oneself that being lonely isn't that important or impactful* |
|  | Acceptance | Everything happens for a reason |
| **Negotiation** | Bargaining | Active attempts to work out a compromise between priorities of person and constraints of situation* |
|  | Priority Setting | Identifying/establishing priorities* |
| **Submission** | Rumination/perseveration | Rumination and/or catastrophizing* |
|  | Intrusive thoughts | Experiencing unwanted thoughts; hard to think other things* |
| **Opposition** | Other blame | Blaming others for feeling lonely* |
|  | Aggression | Shouting* |
| **Delegation** | Maladaptive help seeking | Seeking help from unhelpful sources* |
|  | Complaining/whining | Annoy and pester family |
|  | Self-pity | Feeling sorry for oneself * |
| **Social Isolation** | Concealment | Try not to show |
|  | Avoiding others/withdrawal | Stay alone |
| **Information Seeking** | Reading | Reading about loneliness - trying to learn more about stressful situation/condition, including course, causes, consequences* |
|  | Asking others | Asking others about loneliness * |
| **Example not from current study* | | |

| **Supplemental Table 3**  *Asher Loneliness Scores and ADOS Loneliness Ratings Over Time* | | | | | | | | | | | |
| --- | --- | --- | --- | --- | --- | --- | --- | --- | --- | --- | --- |
| Variable | Age Comparisons | T1: M (SD) | T2: M (SD) | Positive Differences | Ties | Negative Differences | *T* or z | Test Statistic | SE | df or n | *p* |
| ADOS Loneliness-Self | 9 to 19 | n/a | n/a | 5 | 12 | 10 | -1.52 | 34.00 | 17.10 | 27 | 0.13 |
| ADOS Loneliness-Self | 19 to 25 | n/a | n/a | 14 | 23 | 6 | 2.11 | 159.50 | 25.89 | 43 | 0.04 |
| ADOS Loneliness-Others | 19 to 25 | n/a | n/a | 16 | 12 | 6 | 2.65 | 205.00 | 29.60 | 34 | 0.01 |
| Asher Loneliness Score | 17 to 19 | 15.12 (6.06) | 14.57 (5.79) | n/a | n/a | n/a | 0.46 | - | - | 19 | 0.65 |
| Asher Loneliness Score | 19 to 23 | 16.53 (5.72) | 17.77 (6.96) | n/a | n/a | n/a | -1.10 | - | - | 21 | 0.29 |

| **Supplemental Table 4**  *ADOS Loneliness-Self vs Others’ Ratings* | | | | | | | | |
| --- | --- | --- | --- | --- | --- | --- | --- | --- |
| Age | Positive Differences | Ties | Negative Differences | Test Statistic | SE | z | n | *p* |
| 9 | 13 | 12 | 3 | 94.50 | 18.90 | 1.40 | 28 | 0.16 |
| 19 | 26 | 21 | 8 | 491.00 | 57.46 | 3.37 | 55 | <0.001 |
| 25 | 29 | 20 | 5 | 512.50 | 57.28 | 3.75 | 54 | <0.001 |

| **Supplemental Table 5**  *Frequency of Endorsement of Loneliness Related Factors* | | | | | | | |
| --- | --- | --- | --- | --- | --- | --- | --- |
| ***Category*** | ***Subcategory*** | **Age 9** | | **Age 19** | | **Age 25** | |
|  |  | Self  (n = 58) | Others  (n = 29) | Self  (n = 68) | Others  (n = 59) | Self  (n = 59) | Others  (n = 56) |
| Social or Emotional Loneliness | Mentioned social isolation "being alone" | 10 | 2 | 7 | 5 | 7 | 0 |
|  | Mentioned emotional experience of loneliness (e.g., feeling upset, discontent, sad about not having friends) | 0 | 0 | 1 | 0 | 0 | 0 |
| Contextual Factors | Mentioned boredom or not having enough to do | 1 | 0 | 5 | 1 | 1 | 1 |
|  | Mentioned certain days or seasons or situations | 4 | 1 | 7 | 2 | 5 | 0 |
| Types of Relationships | Mentioned friendships or a group of people or community | 2 | 3 | 4 | 7 | 2 | 0 |
|  | Mentioned family members | 3 | 1 | 1 | 1 | 2 | 0 |
|  | Mentioned romantic relationships | 0 | 0 | 3 | 0 | 2 | 1 |
| Individual Factors | Mentioned personal characteristic or disposition (e.g., introversion) | 0 | 0 | 0 | 2 | 3 | 1 |
| Loneliness Changes | Mentioned changes in loneliness or experience of loneliness | 1 | 1 | 5 | 0 | 5 | 0 |
|  | Mentioned difference between others and self in terms of loneliness | 0 | 0 | 1 | 3 | 1 | 6 |
